# Vector analysis of high astigmatism (≥ 2.0 diopters) correction after small-incision lenticule extraction with stringent head positioning and femtosecond laser-assisted laser in situ keratomileusis with compensation of cyclotorsion

**DOI:** 10.1186/s12886-022-02384-0

**Published:** 2022-04-05

**Authors:** Jihong Zhou, Wei Gu, Yan Gao, Guoli He, Fengju Zhang

**Affiliations:** 1 Beijing Tongren Eye Center, Beijing Tongren Hospital, Capital Medical University, No. 1 Dongjiaomin Xiang, Dongcheng District, 100730 Beijing, China; 2 Beijing Aier-Intech Eye Hospital, Beijing, China

**Keywords:** Femtosecond laser-assisted laser in situ keratomileusis, Small-incision lenticule extraction, Astigmatism, Compensation of cyclotorsion, Stringent head positioning

## Abstract

**Background:**

The purpose of this study was to compare the astigmatic correction by vector analysis in patients with high myopic astigmatism after femtosecond laser-assisted laser in situ keratomileusis (FS-LASIK) with cyclotorsion compensation or small-incision lenticule extraction (SMILE) with stringent head positioning.

**Setting:**

Beijing Aier-Intech Eye Hospital, Beijing, China.

**Design:**

A retrospective case series.

**Methods:**

Patients who had correction of myopic astigmatism of 2 diopters (D) or more treated with either FS-LASIK with cyclotorsion compensation or SMILE with stringent head positioning were included. The results of vision and refraction were analyzed and compared between groups with the right eye.

**Results:**

The study enrolled 94 patients (41eyes in an FS-LASIK with compensation of cyclotorsion group and 53 eyes in a SMILE with stringent head positioning control group. The mean preoperative manifest cylinder was -2.65 ± 0.77D in the FS-LASIK group and 2.51 ± 0.56D in the SMILE group (*P* = 0.302). At 12 months, there was no significant between-group difference in uncorrected distance visual acuity (UDVA, *P* = 0.274) and postoperative spherical equivalent (SEQ) (*P* = 0.107). 46.3% and 24.5% of eyes in the FS-LASIK and SMILE groups were within 0.25 D were within 0.25D postoperative cylinder, respectively, and 78% and 66% of eyes in these two groups were within 0.5 D postoperative cylinder (*P* = 0.027, *P* = 0.202). The vector analysis showed comparable between-group target-induced astigmatism (TIA) (*P* = 0.114), surgically induced astigmatism (SIA) (*P* = 0.057), difference vector (DV, *P* = 0.069), and the angle of error (AE) (*P* = 0 .213) values. The index of success (IOS) was 0.18 in the FS-LASIK group and 0.24 in the SMILE group (*P* = 0.024), with a significant difference between the two groups.

**Conclusion:**

FS-LASIK with compensation of cyclotorsion showed a favorable correction of high myopic astigmatism (≥ 2.0 D) compared to SMILE with stringent head positioning at 12 months.

## Introduction

Femtosecond laser-assisted laser in situ keratomileuses (FS-LASIK) was first reported by Ratkay-Traub et al. in 2003 [1]. This procedure has many advantages over a mechanical microkeratome and fewer side effects, such as free caps, irregular flaps, and buttonholes, while complicated with a femtosecond laser makes a flap and then an excimer laser performing the corneal reshaping. Small-incision femtosecond lenticule extraction (SMILE; Carl Zeiss Meditec AG) has become popular in recent years as a new paradigm for myopic refractive errors. Sekundo W et al. [2] presented the first clinical report on the use of SMILE.

in 2008. The SMILE procedure is flapless and preserves more nerve fibers and corneal biomechanical strength. Thus, dry eye and flap-related complications are reduced as compared to FS-LASIK. Many studies have shown that visual and refractive outcomes, safety, efficacy, and predictability are comparable in SMILE and FS-LASIK [3, 4]. However, the precision of astigmatism correction in the SMILE platform is uncertain because of the lack of cyclotorsion control. Khalifa MA et al. [5] reported a trend toward undercorrection and misalignment with SMILE in a 6-month follow-up. Chan et al. [6] showed that the correction with SMILE was less efficacious than with FS-LASIK in those with low to moderate myopic astigmatism but comparable in those with high astigmatism (≥ 3.0 D) in a 3-month follow-up study.

This retrospective study aimed to use vector analysis to compare the efficacy of astigmatic correction in patients with high myopic astigmatism (≥ 2.0 D) after FS-LASIK with compensation of cyclotorsion or SMILE with a stringent head positioning in a long-term (12 months) follow-up study.

### Patients and methods

This study included patients with myopic astigmatism of 2.0 D or more who underwent FS-LASIK or SMILE between September 2013 and June 2020 by the same surgeon (J.H. Zhou).

All data for the study were collected and analyzed by the Institutional Review Board (IRB) of Beijing Aier-Intech Eye Hospital, Beijing, China. The study protocol was approved by the ethics committee and adhered to the Declaration of Helsinki's tenets. All the patients were given information about the risks and benefits of the procedures and signed an informed consent form before surgery.

The inclusion criteria were 18 years or older, myopia and astigmatism were stable, or a minor change in 0.50 D in the least 12 months. Patients with suspected keratoconus on corneal topography, severe dry eyes, ocular inflammation, infection, systemic diseases, immune system diseases, depression, and pregnancy were excluded from the study.

All the patients underwent a comprehensive ophthalmic examination to rule out eye diseases other than myopia and myopic astigmatism. The preoperative evaluation included uncorrected distance visual acuity (UDVA), corrected distance visual acuity (CDVA), and intraocular pressure (IOP) testing using a Nidek NT-510 noncontact tonometer (Nidek Co., Gamagori, Japan); slit-lamp biomicroscopy and subjective manifest and cycloplegic refraction using a comprehensive optometry station (Nidek AOS-1500; Japan); corneal topography and wavefront aberration using an OPD Scan III (Nidek Inc., Tokyo, Japan); and corneal thickness scanning using a Pentacam (Oculus Optikgeräte GmbH; Wetzlar, Germany). Follow-up visits were scheduled at regular intervals (1d, 7d, 1 m, 3 m, 6 m, and 12-month).

Postoperatively, refractions were recorded using an automatic refractometer (NIDEK ARK-510, Japan). UDVA, CDVA, IOP, topography, and wavefront aberration were recorded during the follow-ups. Before surgery, a topical anesthetic (benoxinate hydrochloride 0.4%) was instilled two or three times in the conjunctival fornix of the eye.

The FS-LASIK procedure was performed using an FS200 (WaveLight® AlconSurgical, Fort Worth, TX, USA) femtosecond laser to create the flap (100–110 μm thickness, 8.5- 8.7 mm diameter, and 40° superior hinge), followed by ablation using an EX-500 excimer laser, with pupil-tracking and compensation of cyclotorsion by iris registration. The diameter of the optical zone was employed for 6.00 − 6.7 mm. After laser ablation, the corneal flap was irrigated with a saline solution and repositioned in the stromal bed.

**Fig. 1:**
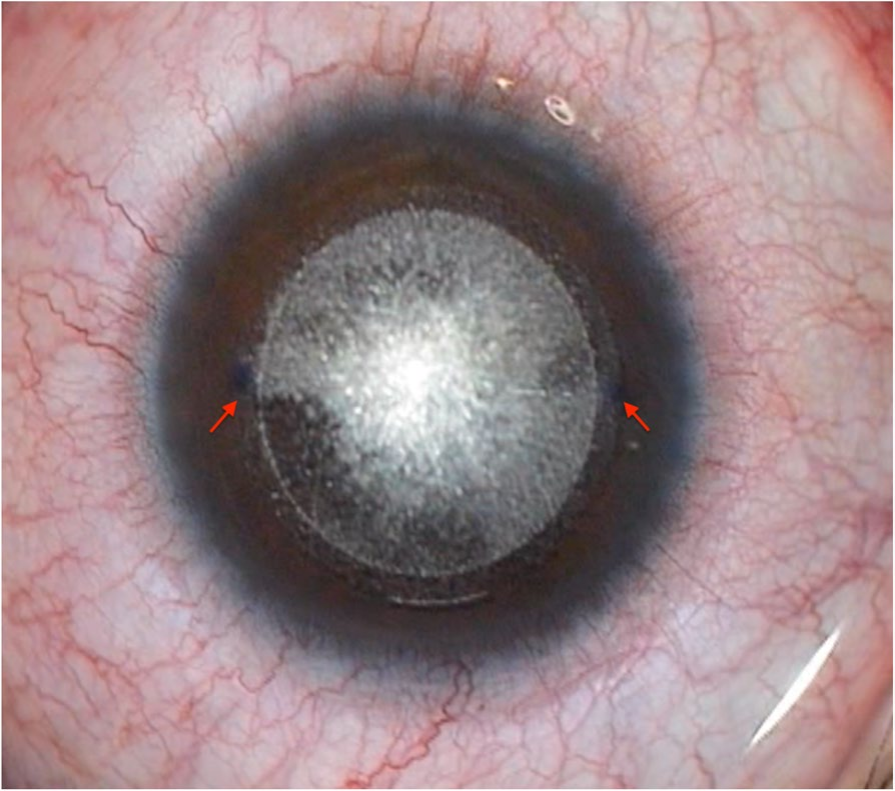
Limbal markings at the 0° to 180° axis in front of a slit-lamp preoperatively

**Fig. 2:**
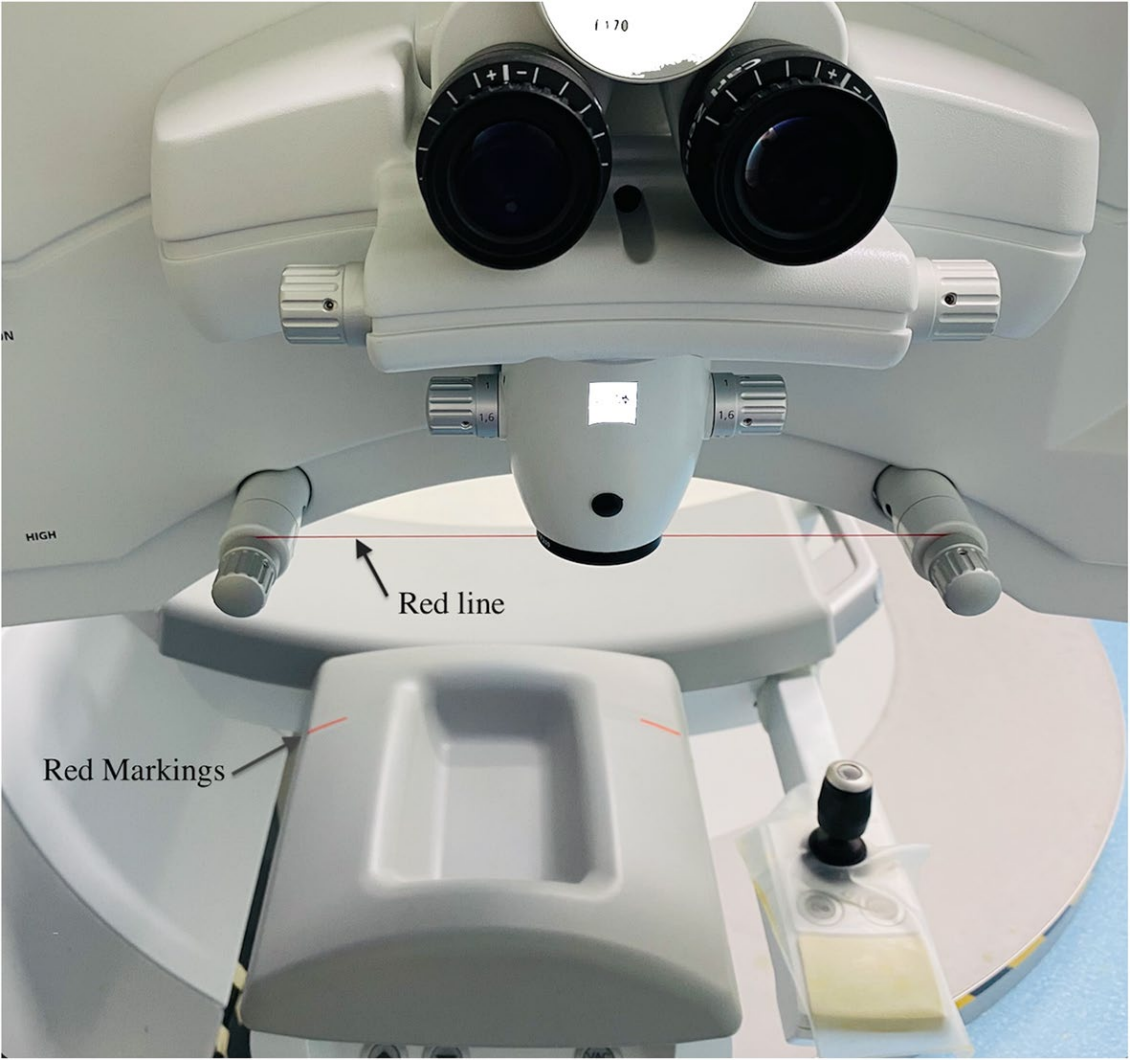
Red markings on the operating bed: The surgical assistant ensured the edge of the patient's earlobes corresponded to the red markings. The red line shows where the surgeon adjusted the patient’s inner and outer canthal angle parallel to the red line

The SMILE procedure was performed using the VisuMax femtosecond laser system (Carl Zeiss Meditec AG, Jena, Germany) with a 500 kHz repetition rate. A small (S) curved interface cone was used in all cases. In all the procedures, the surgeon made manual limbal markings at the 0° to 180° axis before the slit-lamp preoperatively [7] **(**Fig. 1). We appended the red markings on the operation bed and the red line on the slit lamps (Fig. 2). The surgical assistant ensured that the edge of the patient’s earlobes corresponded to the red markings on the operating bed. The surgeon then made the patient’s inner and outer canthal angle parallel to the red line to adjust head cyclotorsion. Finally, the limbal markings were regulated to match the horizontal line of the microscope cross, and suction was applied. The following parameters were applied: cut energy of 105 − 135 nJ, cap and lenticular spot track distance of 4.5 μm, the cap side and lenticular side spot track distance of 1.8 − 2.0 μm, cap thickness of 100 − 130 μm, cap diameter of 7.1 − 7.8 mm, and lenticule diameter of 6.0 − 6.7 mm, depending on the preoperative corneal thickness, pupil size, and the refractive error to be corrected. A small incision was created at 10 or 12 o’clock, with a 2.0 mm side cut. The lenticule was gently separated using a spatula and extracted with a pair of forceps.

### Vector analysis

Vector analysis for astigmatic correction was conducted according to the Alpins method [8]. Three fundamental vectors were examined.

- The target-induced astigmatism (TIA) vector was the vector of the astigmatic correction for which the surgery was attempted.
- The surgically induced astigmatism (SIA) vector was interpreted as the astigmatic achieved by the surgery.
- The difference vector (DV) was defined as the vectorial difference between the TIA and SIA vectors.

We compared outcomes of astigmatism correction from the above three vectors (TIA, SIA, and DV) of various relationships.

- The magnitude of error (ME) was the arithmetic difference between the SIA and TIA.
- The angle of error (AE) was the angle described by the vector of SIA versus TIA. AE was positive if the achieved correction was on an axis counterclockwise (CCW) to where it was intended and negative if the achieved correction was clockwise (CW) to its intended axis. The absolute AE was the angle between the axis of the SIA and TIA [9].
- The correction index (CI) was defined as the SIA divided by the TIA. The value might be preferred, and astigmatism was considered undercorrected if the CI was lower than 1.
- The index of success (IOS) was the DV divided by the TIA. with a value of 0, considered ideal.

### Statistical analysis

Only the right eyes were analyzed to prevent relevance between the two eyes. Data are presented as mean ± standard deviation (SD). An independent t-test was used to estimate continuous variables between the two groups. Linear regression analyses were performed for TIA and SIA (attempted and achieved sphere). An analysis of covariance (ANCOVA) was used to adjust for different preexisting pre-sphere of the baseline. Categorical variables were assessed using Pearson’s chi-squared test. A repeated-measures analysis of variance was applied to compare variables among each follow-up. The data were plotted in nine standard graphs showing the efficacy, predictability, safety, and stability using Microsoft Excel templates designed by the London Vision Clinic [10]. Vector analyses were conducted using AstigMATIC software [11]. A *P*-value of less than 0.05 was considered statistically significant.

## Results

Ninety-four eyes of 94 patients were enrolled. There were 41 eyes in the FS-LASIK group, and 53 eyes were in the SMILE group at the 12 months follow-up. The mean age of the patients in the FS-LASIK and SMILE groups was 27.59 ± 7.47 and 29.79 ± 7.15 years, respectively (*P* = 0.149). Table 1 shows the baseline characteristics of the two groups. The mean of the preoperative manifest cylinder was -2.65 ± 0.77(range: -2.00 to -5.75 D) in the FS-LASIK group and -2.51 ± 0.56 (range: -2.00 to -4.50 D) in the SMILE group (*P* = 0.302). Preoperatively, there was no statistically significant difference in pre-CDVA (*P* = 0.144), sex ratio (*P* = 0.104), central corneal thickness (*P* = 0.729), age (*P* = 0.149), and IOP (*P* = 0.738), whereas there was a statistically significant differences in the manifest sphere (*P* = 0.044) and spherical equivalent (SEQ) (*P* = 0.027) between the two groups. No intraoperative complications occurred in any of the surgeries.

**Table 1:** Preoperative characteristics of eyes in FS-LASIK group and SMILE group

Parameter	FS-LASIK (41Eyes)	SMILE (53 Eyes)	*P*
CDVA-logMAR (Mean ± SD)	0.026 ± 0.04	0.013 ± 0.04	0.144
Sphere (Mean ± SD)	-5.58 ± 2.88	-4.50 ± 1.96	0.044
Cylinder (Mean ± SD)	-2.65 ± 0.77	-2.51 ± 0.56	0.302
SEQ (Mean ± SD)	-6.90 ± 2.76	-5.75 ± 1.94	0.027
CCT (Mean ± SD)	542.66 ± 29.33	544.60 ± 24.91	0.729
Sex (Male: N, %)	14, 20.6	26, 32.5	0.104
Age (Mean ± SD	27.59 ± 7.47	29.79 ± 7.15	0.149
IOP (Mean ± SD)	16.00 ± 2.49	15.83 ± 2.39	0.738

*CDVA* corrected distance visual acuity, *logMAR* logarithm of the minimum angle of resolution, *CCT* central corneal thickness, *IOP* Intraocular pressure, *SEQ* spherical equivalent

*FS-LASIK* femtosecond laser-assisted laser in situ keratomileusis, *SMILE* small-Incision lenticule extraction

### Efficacy and safety

**Fig. 3:**
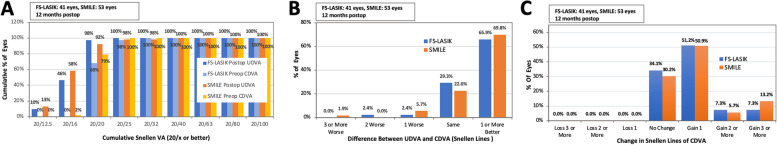
Efficacy and safety of FS-LASIK and SMILE a 12-month. Postoperative UDVA compared with the preoperative CDVA (**A**); postoperative UDVA versus preoperative CDVA (**B**). Change in the lines of postoperative CDVA (**C**). UDVA = uncorrected distance visual acuity, CDVA = corrected distance visual acuity

Table 2 shows the postoperative characteristics of the eyes in the FS-LASIK and SMILE groups. At the 12-month follow-up, there was no significant difference in the UDVA (*P* = 0.274), CDVA (*P* = 0.51), efficacy index (*P* = 0.828), or safety index (*P* = 0.285). As shown in Fig. 3A, 40 eyes (98%) in the FS-LASIK group and 49 eyes (92%) in the SMILE group had a UDVA of 20/20 or better (*P* = 0.274). Figure 3B shows that 95.1% of patients (eyes, *n* = 41) in the FS-LASIK group and 92.5% of patients (eyes, *n* = 53) in the SMILE group had postoperative UDVA the same or better than preoperative CDVA (*P* = 0.600). No eye in the FS-LASIK or SMILE group lost one or more lines in post-CDVA. (Fig. 3C) No corneal complications were detected in any of the patients postoperatively.

**Table 2:** Postoperative characteristics of eyes at 12 months after FS-LASIK and SMILE

	FS-LASIK (41Eyes)	SMILE (53 Eyes)	
Parameter	Mean ± SD	Mean ± SD	P*
UDVA (logMAR)	-0.03 ± 0.10	-0.05 ± 0.08	0.274
CDVA (logMAR)	-0.03 ± 0.09	-0.04 ± 0.08	0.510
Efficacy Index	1.16 ± 0.24	1.17 ± 0.21	0.828
Safety Index	1.19 ± 0.19	1.14 ± 0.26	0.285
Sphere	-0.08 ± 0.60	0.18 ± 0.42	0.028
Cylinder	-0.46 ± 0.32	-0.57 ± 0.40	0.205
SEQ	-0.31 ± 0.63	-0.10 ± 0.49	0.107
Attempted	-6.90 ± 2.77	-4.50 ± 1.96	0.114
Achieved	-6.58 ± 2.77	-4.68 ± 1.96	0.491

^*^Analysis of covariance, pre-sphere was used to adjust for preexisting differences of the baseline. *CDVA* corrected distance visual acuity, *logMAR* logarithm of the minimum angle of resolution, *UDVA* uncorrected distance visual acuity, *SEQ* spherical equivalent, *FS-LASIK* femtosecond laser-assisted laser in situ keratomileusis, *SMILE* small-Incision lenticule extraction

### Predictability

**Fig. 4:**
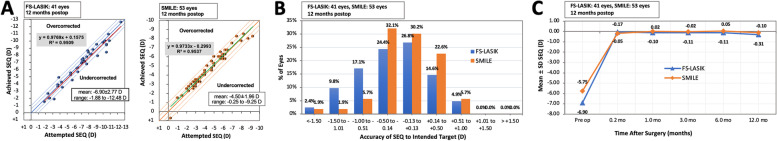
The predictability of spherical equivalent refraction for FS-LASIK and SMILE at 12-month. The attempted vs. achieved spherical equivalent refraction (**A**); Spherical Equivalent Refraction Accuracy (**B**); Spherical Equivalent Refraction Stability (**C**)

At the 12-month follow-up, there was no significant difference in the SEQ (*P* = 0.107) and cylinder (*P* = 0.205), but there was a significant difference in the sphere (*P* = 0.028). Figure 4A shows a scatter plot of the attempted versus achieved SEQ refraction in the FS-LASIK and SMILE groups. Within ± 0.50 D of emmetropia, 27 eyes (65.9%) after FS-LASIK were lower than 45 eyes (84.9%) after SMILE (*P* = 0.031). Thirty-six eyes (87.8%) in the FS-LASIK group and 51eyes (94.4%) in the SMILE group were comparable within ± 1.00D (*P* = 0.248; Fig. 4B). There was no statistically significant difference in the SEQ in the FS-LASIK and SMILE groups throughout the 1 − 12 month postoperative period (*P* = 0.502; Fig. 4C).

### Vector analysis

**Fig. 5:**
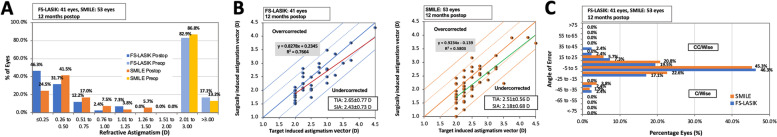
The predictability of cylindrical refraction for FS-LASIK and SMILE at 12-month. Postoperative vs. preoperative refractive astigmatism **(A**)**.** TIA versus SIA (**B**). Refractive Astigmatism AE (**C**). SIA = surgically induced astigmatism; TIA = target-induced astigmatism, AE = angle of error

**Fig. 6:**
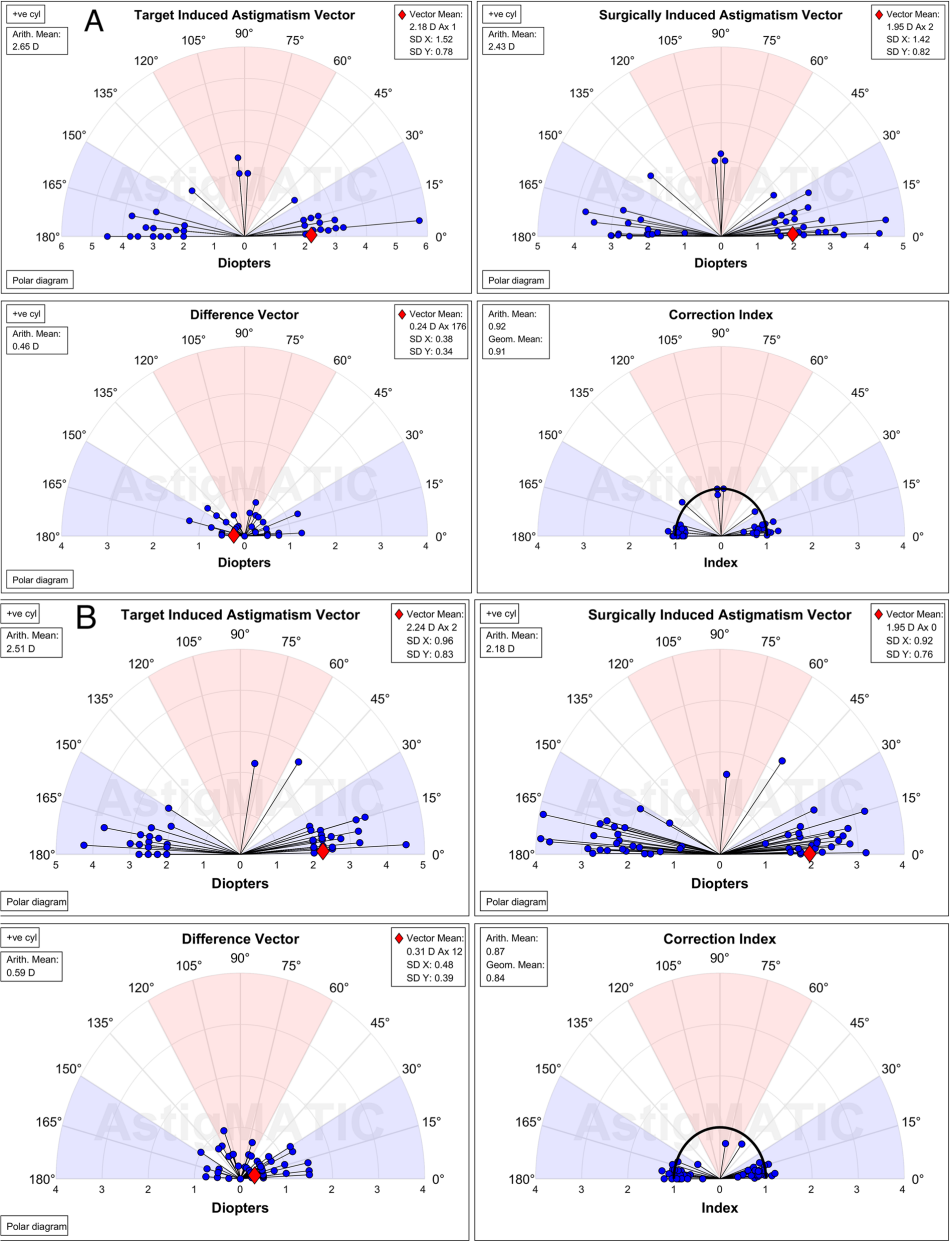
Single angle polar plots of TIA, SIA, DV, and the CI at 12-month after FS-LASIK (A) and SMILE (B). SIA = surgically induced astigmatism; TIA = target-induced astigmatism, DV = difference vector, CI = correction index

Figure 5A displays the amplitude of astigmatism, both postoperative and preoperative. In 19 eyes (46.3%) in the FS-LASIK group and 13 eyes (24.5%) in the SMILE group, it was less than or equal to 0.25D cylinder (*P* = 0.027). Correspondingly, 32 eyes (78%) and 35 eyes (66%) had a postoperative cylinder ≤ 0.50 D (*P* = 0.202), and 38 eyes (92.7%) and 48 eyes (90.6%) had a postoperative cylinder ≤ 1.00D (*P* = 0.715). Figure 5B presents a scatter plot of TIA versus SIA after 12 months. Figure 5C shows the astigmatism AE for the two procedures (*P* = 0.231). Figure 6 shows single-angle polar plots, with a vector mean of TIA, SIA, DV, and CI for FS-LASIK (A) and SMILE (B) at the 12-month follow-up. The scope in the arithmetic mean TIA was 2.00 to 5.75 D in the FS-LASIK group and 2.00 to 4.50 D in the SMILE group (*P* = 0.114). There was no significant difference in SIA (*P* = 0.057), ME (*P* = 0.425), DV (*P* = 0.069), CI (*P* = 0.232) between FS-LASIK and SMILE. However, the IOS was significantly higher in the SMILE group than in the FS-LASIK group (*P* = 0.024). Table 3 shows a comparative analysis of the vector astigmatic results.

**Table 3:** Vector analysis results of astigmatic correction at 12 months after FS-LASIK and SMILE

	FS-LASIK (41)	SMILE (53)	
Parameter	Mean ± SD	Mean ± SD	P*
TIA(D) arithmetic mean	2.65 ± 0.77	2.51 ± 0.56	0.114
Vector mean	2.18@1^º^	2.24@2^º^	
TIAx	-2.18 ± 1.52	-2.24 ± 0.96	0.876
TIAy	-0.09 ± 0.78	-0.15 ± 0.83	0.763
SIA (D) arithmetic mean	2.43 ± 0.73	2.18 ± 0.68	0.057
Vector mean	1.95@2^º^	1.95@0^º^	
SIAx	-1.95 ± 1.42	-1.95 ± 0.92	0.926
SIAy	-0.12 ± 0.82	-0.02 ± 0.76	0.536
ME (D)	-0.22 ± 0.38	-0.33 ± 0.44	0.425
DV (D) arithmetic mean	0.46 ± 0.32	0.59 ± 0.35	0.069
Vector mean	0.24@176^º^	0.31@12^º^	
DV-x	-0.24 ± 0.38	-0.29 ± 0.48	0.842
DV-y	0.03 ± 0.34	-0.13 ± 0.39	0.051
CI	0.92 ± 0.14	0.87 ± 0.19	0.232
AE (degree)	2.14 ± 12.05	-1.13 ± 11.18	0.213
AE_ABS_	7.76 ± 9.40	8.11 ± 7.70	0.931
IOS	0.18 ± 0.13	0.24 ± 0.13	0.024

^*^Analysis of covariance, pre-sphere was used to adjust for preexisting differences of the baseline

*TIA* Target induced astigmatism vector

*SIA* Surgically induced astigmatism vector

*ME* Magnitude of error, the arithmetic difference between the magnitudes of the SIA and TIA

*CI* Correction Index (SIA/TIA)

*AE* Angle of error

*AE*_*ABS*_ Absolut angle of error

*DV* Difference Vector

*IOS* Index of success (DV/TIA)

*FS-LASIK* femtosecond laser-assisted laser in situ keratomileusis

*SMILE* small-Incision lenticule extraction

## Discussion

This study proved that SMILE and FS-LASIK had comparable efficacy and safety in correcting high myopic astigmatism. UDVA of 20/25 or better was 98% after SMILE and 100% after FS-LASIK, and CDVA was not lost in any eye in both groups. Chan et al. [9] also reported no difference in outcomes of vision and refraction after FS-LASIK and SMILE after 3 months. Liu et al. [12] found similar results in a comparative study. The current study showed that predictability within ± 0.50 D was 85% and 66% of SEQ after SMILE and FS-LASIK (*P* = 0.031), respectively, similar to a study by Han et al. [13]. The predictability of SEQ after SMILE was better than after FS-LASIK. In addition, myopia was higher after FS-LASIK than after SMILE. There was no significant between-group difference in the stability of postoperative SEQ. Shift forward myopia was fewer after 12 months for FS-LASIK and SMILE, parallel with previous results [13, 14].

We used the Alpins method to assess astigmatic correction (≥ 2.0 D) [8]. At the 12-month follow-up, our results revealed no statistically significant differences between the FS-LASIK and SMILE groups in any of the measured parameters (TIA, SIA, ME, DV, CI, and AE), other than the IOS. To the best of our knowledge, this is the first study to compare long-term outcomes (12 months) in patients with high myopic astigmatism (≥ 2.0 D) by vector analysis after FS-LASIK with compensation of cyclotorsion by iris registration and SMILE (with a stringent head positioning and manual limbal marking to correct.

Based on our results, mean preoperative cylinder of -2.65D, TIA of 2.65D in FS-LASIK, -2.51 D, and TIA of 2.51 D in the SMILE group were estimated. Postoperatively undercorrection of astigmatism was observed, with a mean cylinder of -0.46 and -0.57 D, ME of -0.22 and -0.33 D, and CI of 0.92 and 0.87 in the FS-LASIK and SMILE groups, respectively after 12 months. Zhang et al. [15] reported a CI of 0.94 in wavefront-guided FS-LASIK and 0.88 in SMILE by analyzing a mean preoperative cylinder of TIA of 2.48 to 2.65D after 3 months. The results of previous studies showed a certain extent of undercorrection after both surgical procedures [6, 9, 15, 16].

The axis of the cylinder aligned is critical during the treatment. Ganesh et al. [17] accomplished manual cyclotorsion compensation directed by preoperative limbal marking and applied a 10% overcorrection nomogram. In their study, the mean preoperative cylinder was -2.48 D, TIA was 2.19 D. The postoperative cylinder was -0.31 D, and the ME, CI, and IOS were -0.149 D, 0.93, and 0.14, respectively, 3 months post-treatment. Pedersen et al. [18] suggested that a nomogram adjustment by 10% in the magnitude of astigmatism correction could be beneficial. Our study applied only about a 10% overcorrection nomogram for the sphere, not for the cylinder in SMILE, and long-term follow-up might be the extra favorite undercorrection.

According to a previous study, myopic ablation, related to corneal epithelial remodeling, increases after one month and up to 1 year after FS-LASIK [19]. Our results were consistent with the previous that high astigmatic correction by FS-LASIK seemed better than SMILE, although there was no statistically significant difference in DV, ME, and CI. At the 12-month follow-up, the average DV was 0.24 @ 176° in the FS-LASIK and 0.31 @ 12° in the SMILE group. The average AE value was positive (2.14) after FS-LASIK and negative (-1.13) after SMILE. The absolute AE found in the current study was 7.76° in the FS-LASIK group and 8.11° in the SMILE group, comparable to that found by Pedersen et al. [18], who reported an AE of 0.34 and an absolute mean AE of 8.94. Zhang et al. [20] reported similar findings with an AE of -3.04 and an absolute mean AE of 6.08 after SMILE in a 12-month follow-up study. In our study, the mean absolute AE value was slightly higher after SMILE than after FS-LASIK, but the difference was not statistically significant. When calculated by vector analysis, the proportion of loss of flattening effect (FE) was 1.5% when the treatment was 5°misaligned, 13.4% when 15°, and 50% when 30° [21]. In our study, the residual cylinder was 0.25 D or less in 46.3% of eyes in the FS-LASIK group and 24.5% of eyes in the SMILE group after high astigmatic correction (≥ 2.0 D) (*P* = 0.027) at the 12-month follow-up. In a study by Kanellopoulos [22], 82% of eyes after LASIK and 50% of eyes after SMILE had a residual cylinder of 0.25 D or less in a 3-month follow-up, with correction of astigmatism (≥ 1.5 D). The difference between the two studies might be attributed to the disparity in the degree of astigmatism and the shorter follow-up times. FS-LASIK with compensation of cyclotorsion had better predictability of astigmatic correction.

In this study, we took three steps to control the cyclotorsion compensation and others in the SMILE procedure: preoperative marking on the limbal, a stringent head positioning, and limbal marking matching the horizontal line of the microscope cross. We did not gently rotate the cone of the eye when starting suction, as the manual limbal marking method might introduce varying degrees from 3.8 to 6.0° inherently [23]. Prickett et al. [24] observed that most of the rotations previously attributed to torsional components were probably due to noncyclotorsion components, such as postural misalignments. Chan et al. [9] also emphasized that the position of the head to both eyes aligns along an imagined horizontal line without the manual limbal marking; the further reason might be 86% of eyes for high astigmatism within 5° or less cyclotorsion in a survey by Ganesh et al. [17]. Manual limbal marking and stringent head positioning both affirmed a safe, feasible, and effective strategy to perfect the results of high astigmatic correction with SMILE.

Shen et al. [7] compared manual limbal markings versus iris-registration systems in LASIK and concluded that manual limbal marking was a safe alternative when automated systems were unavailable. Zhao et al. [16] also showed that wavefront-guided FS-LASIK and optimized SMILE achieved similar outcomes in terms of astigmatism correction. Various cyclotorsion alignment methods have been used in SMILE to improve astigmatism correction [16, 17, 25, 26]. In contrast, the system of iris registration for the VisuMax platform is warranted in the future to enhance its capability in astigmatic correction.

In the present study, the efficacy of SMILE with stringent head positioning in correcting astigmatism was lower than that of FS-LASIK with compensation of cyclotorsion using an iris-registration system. An IOS of 0.00 indicates complete success in astigmatism treatment. In contrast, a value of 1.00 denotes no improvement compared to the preoperative status, and a value greater than 1.00 indicates a deterioration in astigmatism [8]. In previous studies, the IOS varied from 0.07 to 0.17 3 to 6 months after FS-LASIK and from 0.09 to 0.15 in the same period after SMILE [9, 15]. In our study, the IOS was 0.18 after FS-LASIK and 0.24 after SMILE at the 12-month follow-up, with a significant difference between the two groups. The higher IOS in our study compared to that reported in the literature might be due to the longer follow-up time in our research. Ivarsen et al. [27] also demonstrated a tendency for greater undercorrection over time, with higher degrees of astigmatic correction. According to a previous study, over time, higher undercorrection with high astigmatic correction might be associated with corneal epithelial remodeling [19].

### Limitations

A limitation of this study was the small sample size of high astigmatic correction. Varma R et al. [28] reported that the prevalence of high astigmatism, defined as over 2.25 D, was only 3.7% (3.1%-4.3%) in Chinese American adults. At the start of our study, we enrolled 840 right eyes of 840 patients for high astigmatism (≥ 2.0 diopters); only 94 right eyes (11.2%) finished the process at the 12-month follow-up. This study used the right eye to avoid correlations between the right or left eyes but ignored the torsions difference for the two eyes. At each follow-up, we used an automatic optometer to detect postoperative refraction. Eye accommodation might impact for troublesome to acquire the manifested and cycloplegic refraction. However, due to the adult age of the patients enrolled in our study, eye accommodation might have less effect on the results. Pesudovs [29] proved excellent agreement between autorefraction and subjective refraction. The current study had a retrospective design. Thus, the potential selection bias could not be excluded as in a randomized clinical trial. Prospective, randomized clinical trials with larger sample sizes of both eyes are needed.

## Conclusions

In conclusion, SMILE with stringent head positioning was less effective than FS-LASIK with compensation of cyclotorsion in terms of high astigmatic correction (≥ 2.0 D) after 12-months. Further study should improve SMILE with an eye-tracking system and cyclotorsion compensation to correct astigmatism accurately in the future.

## Data Availability

The datasets generated and analyzed during the current study are not publicly available (due to the data do not have consent from all patients to publish, it will need to be de-identified) but are available from the corresponding author on reasonable request.

## Abbreviations

FS-LASIK: Femtosecond laser-assisted laser in situ keratomileusis
SMILE: Small-Incision lenticule extraction
UDVA: Uncorrected distance visual acuity
CDVA: Corrected distance visual acuity
logMAR: Logarithm of the minimum angle of resolution
CCT: Central corneal thickness
IOP: Intraocular pressure
SEQ: Spherical equivalent
SIA: Surgically induced astigmatism
TIA: Target-induced astigmatism
AE: Angle of error
DV: Difference vector
ME: Magnitude of error
CI: Correction index
IOS: Index of success
